# Bmp2 regulates Serpinb6b expression via cAMP/PKA/Wnt4 pathway during uterine decidualization

**DOI:** 10.1111/jcmm.15372

**Published:** 2020-05-11

**Authors:** Hai‐Fan Yu, Lian‐Wen Zheng, Zhan‐Qing Yang, Yu‐Si Wang, Ji‐Cheng Huang, Shu Liu, Zhan‐Peng Yue, Bin Guo

**Affiliations:** ^1^ College of Veterinary Medicine Jilin University Changchun China; ^2^ Reproductive Medical Center the Second Hospital of Jilin University Changchun China

**Keywords:** Bmp2, cAMP‐PKA‐Wnt4 pathway, decidualization, Serpinb6b, uterine stromal cell

## Abstract

Serpinb6b is a novel member of Serpinb family and found in germ and somatic cells of mouse gonads, but its physiological function in uterine decidualization remains unclear. The present study revealed that abundant Serpinb6b was noted in decidual cells, and advanced the proliferation and differentiation of stromal cells, indicating a creative role of Serpinb6b in uterine decidualization. Further analysis found that Serpinb6b modulated the expression of Mmp2 and Mmp9. Meanwhile, Serpinb6b was identified as a target of Bmp2 regulation in stromal differentiation. Treatment with rBmp2 resulted in an accumulation of intracellular cAMP level whose function in this differentiation program was mediated by Serpinb6b. Addition of PKA inhibitor H89 impeded the Bmp2 induction of Serpinb6b, whereas 8‐Br‐cAMP rescued the defect of Serpinb6b expression elicited by Bmp2 knock‐down. Attenuation of Serpinb6b greatly reduced the induction of constitutive Wnt4 activation on stromal cell differentiation. By contrast, overexpression of Serpinb6b prevented this inhibition of differentiation process by Wnt4 siRNA. Moreover, blockage of Wnt4 abrogated the up‐regulation of cAMP on Serpinb6b. Collectively, Serpinb6b mediates uterine decidualization via Mmp2/9 in response to Bmp2/cAMP/PKA/Wnt4 pathway.

## 1 INTRODUCTION

In mice, blastocyst attaches to uterine epithelium at day 4.5 of pregnancy. With the initiation of attachment, uterine stromal cells surrounding the implanting embryo begin to undergo extensive proliferation and subsequently differentiate into decidual cells, a process known as decidualization which is a prerequisite for continued embryonic development and successful pregnancy.^1^, ^2^ It has been well established that defects in decidualization can lead to adverse outcome, such as foetal embryo miscarriage, growth retardation, recurrent spontaneous abortion and early pregnancy loss.^3^, ^4^ Numerous studies have demonstrated that multiple growth factors, transcription factors and signalling molecules participate in the regulation of decidualization. Among these regulators, bone morphogenetic protein 2 (Bmp2), a members of the transforming growth factor β (TGF‐β) superfamily, has received increasing attention in the field of female reproduction.^2^, ^5^, ^6^ Mice deficiency in uterine Bmp2 is infertile and incapable of undergoing the decidual reaction.^5^ In human stromal cells, administration of recombinant Bmp2 protein (rBmp2) significantly advanced the differentiation process, whereas knock‐down of Bmp2 efficiently blocked this program.^5^, ^7^ Further analysis found that physiological function of Bmp2 in decidualization was mediated by wingless‐related murine mammary tumour virus integration site 4 (Wnt4).^7^, ^8^ However, the downstream regulatory mechanisms by which Bmp2‐Wnt4 pathway controls stromal differentiation remain to be further elucidated.

Serine peptidase inhibitors (Serpins) are the largest and most broadly distributed family of protease inhibitors and essential for regulating diverse biological processes, including immune function, apoptosis, blood clotting, tumorigenesis and cancer metastasis.^9^ Serpins in clade B consists of intracellular serpins, and its physiological functions have been widely studied in programmed cell death, autoimmunity, inflammatory and cancer progression.^10^, ^11^, ^12^, ^13^, ^14^, ^15^ Serpin, clade B, member 6b (Serpinb6b, also named as serine protease inhibitor), has been identified as a novel member of Serpinb family. Only report demonstrated that Serpinb6b was expressed in germ and somatic cells of mouse gonads,^16^ but there is almost no research to elucidate its biological function. Based on the preliminary microarray analysis,^17^ Serpinb6b is up‐regulated in polyploid decidual cells as compared to non‐polyploid cells, implying an involvement of Serpinb6b in decidualization. The present study uncovers a novel insight into the role of Serpinb6b in uterine decidualization and identifies it as a downstream target of cAMP/PKA/Wnt4 pathway in response to Bmp2.

## 2 MATERIALS AND METHODS

### 2.1 Ethics approval

All animal experiment procedures were approved by the Committee for the Ethics on Animal Care and Use of Jilin University (SY201905031).

### 2.2 Animal treatment

Matured Kunming white mice were caged in a controlled environment with a cycle of 14L:10D. Adult female mice were mated with fertile or vasectomized males to induce pregnancy or pseudopregnancy (day 1 = day of vaginal plug). Model of artificial decidualization was induced by infusing 25 µL of sesame oil into one uterine horn on day 4 of pseudopregnancy, while the contralateral uninjected horn served as a control. The uteri were collected at 48, 72 or 96 hours after oil infusion.

### 2.3 In situ hybridization

In situ hybridization was performed as described previously.^18^ Briefly, cRNA probes were labelled with Digoxigenin according to RNA labelling kit (Roche). After fixation and hybridization with above probes, frozen sections were incubated with sheep anti‐digoxigenin antibody conjugated to alkaline phosphatase (1:5000; Roche) and then stained with 5‐bromo‐4‐chloro‐3‐indolyl phosphate (BCIP, 0.4 mmol/L)/nitroblue tetrazolium (NBT, 0.4 mmol/L) buffer. The sense probe was also hybridized and served as a negative control.

### 2.4 Real‐time PCR

After total RNAs were isolated and reverse‐transcribed into cDNA, real‐time PCR was performed using FS Universal SYBR Green Real Master (Roche) on LightCycler 96 Real Time Detection System. After analysis using the 2^−ΔΔCt^ method, data were normalized to Gapdh expression. Primer sequences for real‐time PCR were listed in Table 1.

**TABLE 1:** Primers used in this study

Gene	Sequence of Forward Primer	Sequence of Reverse Primer	Application
Serpinb6b	TGGATAAATGCAGTGGCAAA	TTGCACAGGTTTCACCACAT	In situ hybridization
Serpinb6b	TGGTCTTCATGGGGGCAAAA	TGTGTGCCAGTCTTGTTCGT	Real‐time PCR
Ccna1	GCCCGACGTGGATGAGTTT	AGGAGGAATTGGTTGGTGGTT	Real‐time PCR
Ccnb1	CTGAGCCTGAGCCTGAACCT	AGCCCCATCATCTGCGTCT	Real‐time PCR
Ccnb2	GCTAGCTCCCAAGGATCGTC	CTGCAGAGCTGAGGGTTCTC	Real‐time PCR
Ccnd1	GGGATGTGAGGGAAGAGGTGA	GCAGCGAAAACAACGTGAAA	Real‐time PCR
Ccnd3	CCTCCTACTTCCAGTGCGTG	GGCAGACGGTACCTAGAAGC	Real‐time PCR
Ccne1	AATGGATGGTTCCGTTCGC	TGGGTCTGGATGTTGTGGG	Real‐time PCR
Cdk1	CTGGGCACTCCTAACAACGAAG	TCCAAGCCGTTCTCGTCCAG	Real‐time PCR
Cdk2	ACAGGGCAAGGTGAAAGAC	AGGAGGACGGCAATGAGG	Real‐time PCR
Cdk4	GTGGCTGAAATTGGTGTCGG	TAACAAGGCCACCTCACGAA	Real‐time PCR
Cdk6	TCCTGCTCCAGTCCAGCTAT	CCACGTCTGAACTTCCACGA	Real‐time PCR
Prl8a2	AGCCAGAAATCACTGCCACT	TGATCCATGCACCCATAAAA	Real‐time PCR
Prl3c1	GCCACACGATATGACCGGAA	GGTTTGGCACATCTTGGTGTT	Real‐time PCR
Mmp2	GGATACCCCAAGCCACTGAC	ACGACGGCATCCAGGTTATC	Real‐time PCR
Mmp9	GCACCTCCCACTATGTGTCC	CAAGGATTGTCTGCCGGACT	Real‐time PCR
Alk2	TTCGCCGGAGAAGGACTC	GCTGCATAGCAGATTTGGGC	Real‐time PCR
Bmp2	CGCAGCTTCCATCACGAA	CGCAGCTTCCATCACGAA	Real‐time PCR
Wnt4	TCGTCTTCGCCGTGTTCT	CTGCACCTGCCTCTGGAT	Real‐time PCR
Gapdh	GCCTTCCGTGTTCCTACCC	TGCCTGCTTCACCACCTTC	Real‐time PCR

### 2.5 Stromal cell treatment

As previously described,^19^ stromal cells from day 4 of pregnancy were isolated and cultured with DMEM‐F12 containing 2% charcoal‐treated FBS (Gibco). Afterwards, cells were treated with rBmp2 (100 ng/mL; Peprotech) or cAMP analogue 8‐bromoadenosine‐cAMP (8‐Br‐cAMP, 500 μmol/L; Sigma) with/without H89 (10 μmol/L; Cell Signaling Technology). H89 was dissolved in DMSO, while 8‐Br‐cAMP and rBmp2 were dissolved in PBS. Controls received the vehicle only.

### 2.6 RNA interference

Stromal cells were transfected with siRNAs using Lipofectamine™ 3000 (Invitrogen). After transfection with Alk2, Bmp2, Wnt4 or Serpinb6b siRNA, cells were collected at 48 hours in the absence or presence of rBmp2 or 8‐Br‐cAMP. The corresponding siRNA sequences were list in Table 2.

**TABLE 2:** The siRNAs used in this study

Gene	Sense	Anti‐sense
Serpinb6b	GCUUCCCUAUGUUGGCAAUTT	AUUGCCAACAUAGGGAAGCTT
Alk2	GGGCUGCUUUCAGGUUUAUTT	AUAAACCUGAAAGCAGCCCTT
Bmp2	GUUUCCAGCACCGAAUUAATT	UUAAUUCGGUGCUGGAAACTT
Wnt4	CGUGCAACAAGACAUCUAATT	UUAGAUGUCUUGUUGCACGTT
Negative Control	UUCUCCGAACGUGUCACGUTT	ACGUGACACGUUCGGAGAATT

### 2.7 Plasmid construction and transfection

Full‐length cDNA fragments were amplified using the following primers: Serpinb6b (5′‐GAATTC [EcoR V] ATCATGGATCCACTGCTGG and 5′‐AAGCTT [Hind III] TCATGGGGAGGAGAACCGACC) and Wnt4 (5′‐GAATTC [EcoR I] ATGAGCCCCCGTTCGTGCCT and 5′‐AAGCTT [Hind III] TCACCGGCACGTGTGCATC). After purification and clone into pGEM‐T vector, these vectors were cut by corresponding enzymes. Then, the fragments were ligated into pcDNA3.1 with T4 ligase (Promega) to construct Serpinb6b and Wnt4 overexpression plasmids. An empty pcDNA3.1 expression vector was served as control. After transfection with control plasmid or overexpression plasmid using Lipofectamine™ 3000 (Invitrogen), stromal cells were collected for 48 hours.

### 2.8 Cell proliferation

After transfection with Serpinb6b overexpression plasmid or siRNA, stromal cells were cultured for 48 hours, added MTS reagent (Promega) into each well and then incubated for another 2‐3 hours. Absorbance was measured at 490 nm using a multi‐mode reader.

### 2.9 Intracellular cAMP measurement

The intracellular cAMP levels were measured as described previously.^19^ After transfection with Alk2 siRNA and addition of rBmp2, stromal cells were cultured for 48 hours. After removing the media, cells were incubated with the Induction Buffer containing 100 μmol/L 4‐(3‐butoxy‐4‐methoxy‐benzyl) imidazolidone and 500 μmol/L IBMX, and then tested using cAMP‐Glo™ Assay (Promega). Luminescence was measured with a Cell Imaging Multi‐Mode Reader.

### 2.10 Statistics

All the experiments were independently repeated at least three times. Comparison of two groups was made by independent samples t test. And the multiple comparisons were tested with one‐way ANOVA. Both statistical analyses were performed using SPSS19.0 software program (SPSS Inc). *P* < .05 was considered statistically significant.

## 3 RESULTS

### 3.1 Serpinb6b mRNA expression during decidualization

**FIGURE 1:**
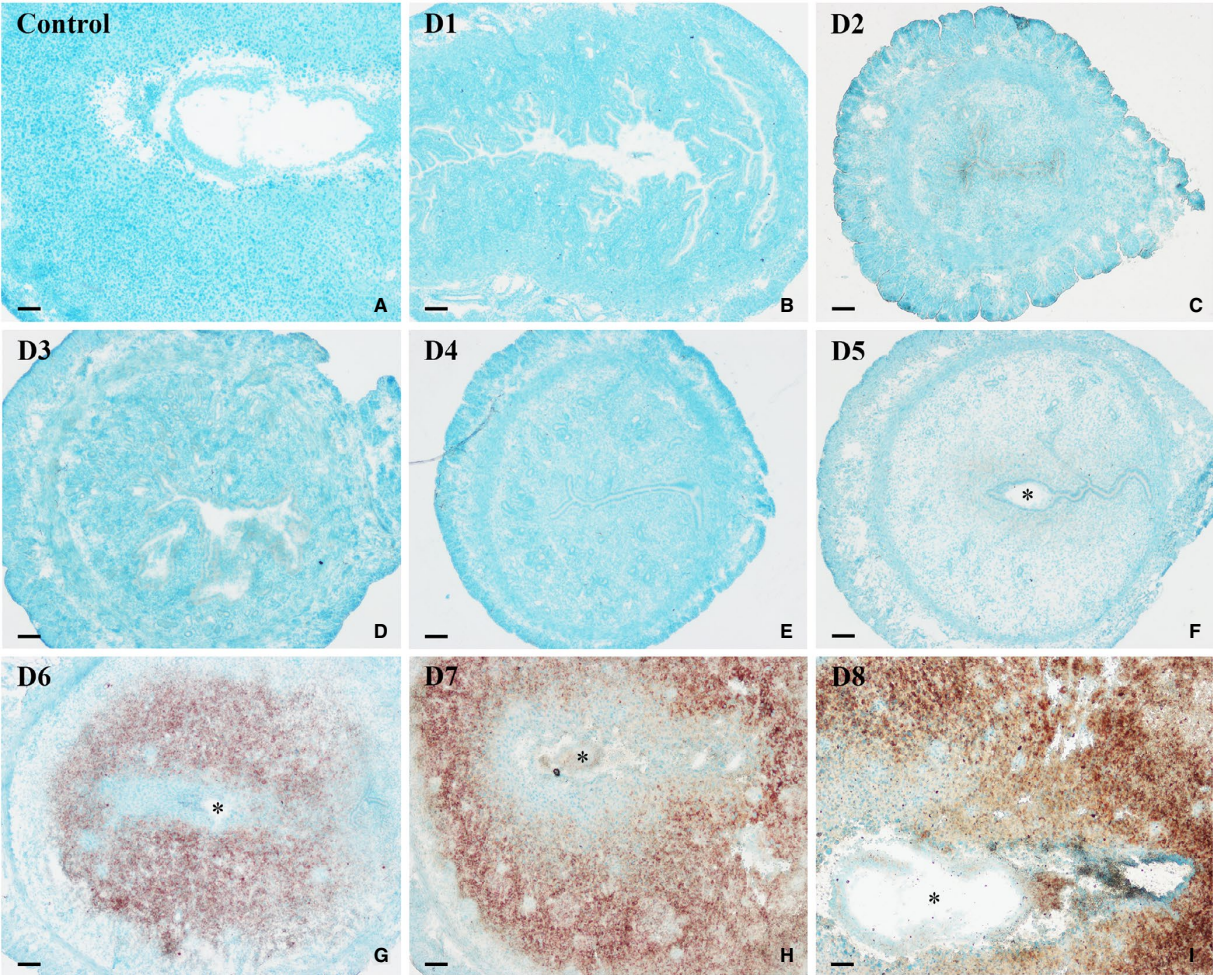
In situ hybridization analysis of Serpinb6b expression in mouse uteri during early pregnancy on days 1 (B), 2 (C), 3 (D), 4 (E), 5 (F), 6 (G), 7 (H) and 8 (I). No hybridization signals were detected in mouse uterus when Serpinb6b sense probe was used to replace its antisense probe as a negative control (A). Asterisks indicate embryo. Scale bar = 60 μm

**FIGURE 2:**
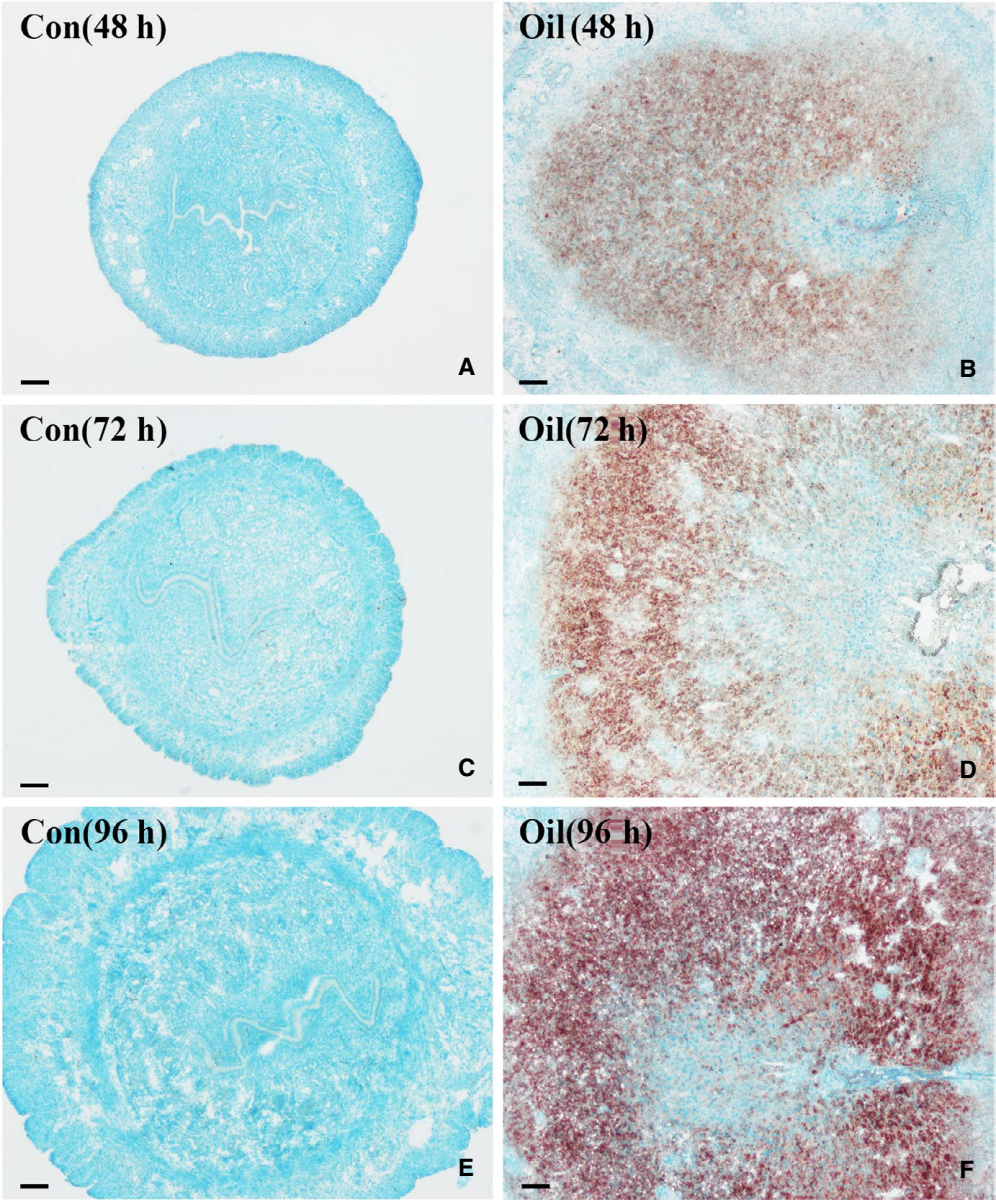
In situ hybridization analysis of Serpinb6b expression under artificial decidualization. Con, uninjected uterine horn; Oil, oil‐induced decidualization

To explore the correlation between Serpinb6b and uterine decidualization, we examined its expression in the uteri during early pregnancy and artificial decidualization. The results showed that Serpinb6b mRNA signal was extremely faint in the uterus from days 1 to 5 of pregnancy (Figure 1B‐F). Nevertheless, from days 6 to 8, abundant Serpinb6b expression was detected in decidual cells (Figure 1G‐I). When Serpinb6b antisense probe was replaced by its sense probe, there was no detectable signal in day 8 uterus (Figure 1A). Real‐time PCR analysis also exhibited an expected elevation on days 6 to 8 of pregnancy (Figure S1A). Meanwhile, a high level of Serpinb6b mRNA signal was seen in the decidualizing stromal cells under artificial decidualization, while no signal was found in the uninjected control uteri (Figure 2A‐F). Consistently, real‐time PCR result revealed that Serpinb6b expression was time‐dependently up‐regulated followed by oil infusion (Figure S1B).

### 3.2 Role of Serpinb6b during decidualization

**FIGURE 3:**
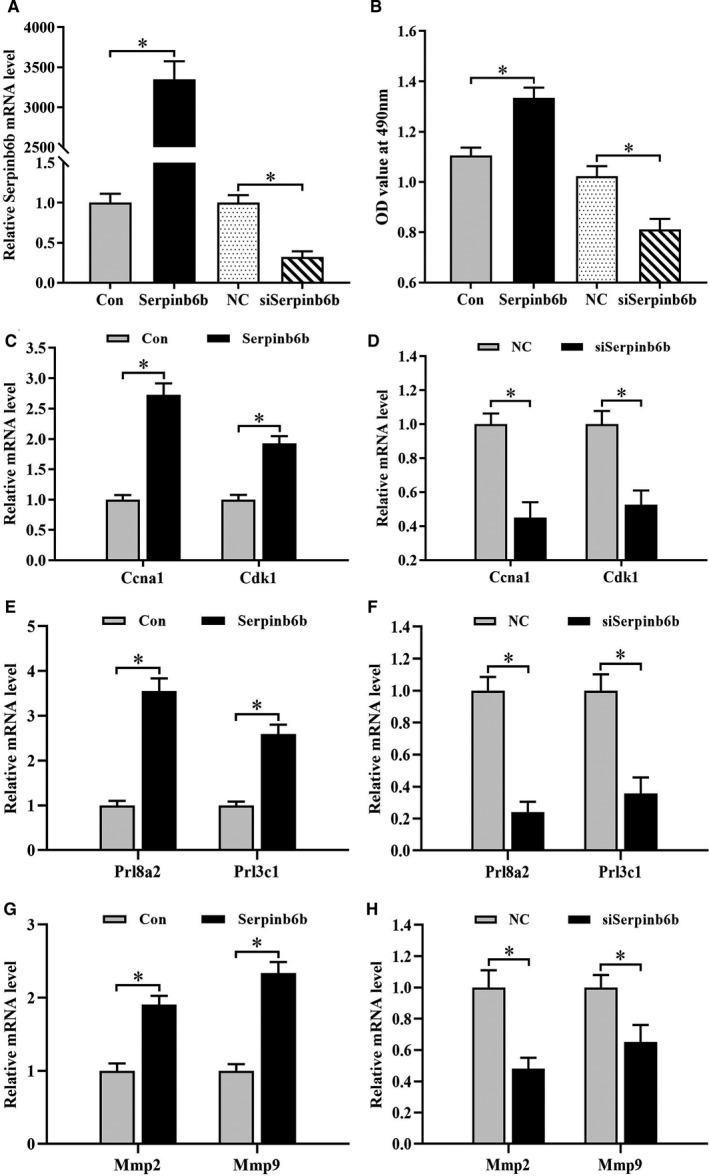
Effects of Serpinb6b on the proliferation and differentiation of uterine stromal cells as well as Mmp2/9 expression. A, Real‐time PCR detection of Serpinb6b expression after transfection with Serpinb6b overexpression plasmid or siRNA. B, Effects of Serpinb6b overexpression or silencing on the proliferation of uterine stromal cells. C and D, Effects of Serpinb6b overexpression or silencing on the expression of Ccna1 and Cdk1. E and F, Effects of Serpinb6b overexpression or silencing on the expression of Prl8a2 and Prl3c1. G and H, Effects of Serpinb6b overexpression or silencing on the expression of Mmp2 and Mmp9. Con, empty pcDNA3.1 vector; Serpinb6b, Serpinb6b overexpression plasmid; NC, negative control; siSerpinb6b, Serpinb6b siRNA. *denote significance (*P* < .05)

To determine the function of Serpinb6b in uterine decidualization, we analysed its influences on stromal cell proliferation and differentiation which are two crucial steps for decidualization.^2^ After transfection with Serpinb6b overexpression plasmid which significantly up‐regulated its mRNA level, proliferation activity of stromal cells was obviously enhanced (Figure 3A,B). On the contrary, knock‐down of Serpinb6b by siRNA, which exhibited the obviously repressive role in corresponding mRNA expression, weakened stromal cell proliferation (Figure 3A,B). Further data showed that Serpinb6b overexpression resulted in a marked up‐regulation in the expression of cyclin A1 (Ccna1) and cyclin‐dependent kinase 1 (Cdk1), whereas silencing of Serpinb6b had the opposite effects (Figure 3C,D). However, no obvious change in the expression levels of Ccnb1, Ccnb2, Ccnd1, Ccnd3, Cdk2, Cdk4 and Cdk6 was observed after transfection with Serpinb6b overexpression plasmid or siRNA (Data not shown).

The influence of Serpinb6b on the differentiation of stromal cells was determined by real‐time PCR analysis of the expression of prolactin family 8, subfamily a, member 2 (Prl8a2) and prolactin family 3, subfamily c, member 1 (Prl3c1), which are well‐established differentiation makers for decidualization in mice.^19^, ^20^ In uterine stromal cells, overexpression of Serpinb6b significantly stimulated the expression levels of Prl8a2 and Prl3c1. In contrast, the expression of Prl8a2 and Prl3c1 was remarkably decreased in Serpinb6b siRNA‐transfected stromal cells (Figure 3E,F).

### 3.3 Regulation of Serpinb6b on the expression of Mmp2 and Mmp9

To explore a potential mechanism for Serpinb6b in uterine decidualization, we analysed its regulation on the expression of matrix metallopeptidase 2 (Mmp2) and Mmp9 whose expression in the decidua was overlapped with that of Serpinb6b.^21^ The results showed that after introduction of Serpinb6b overexpression plasmid, stromal cells exhibited obvious elevation for mRNA levels of Mmp2 and Mmp9, while silencing of Serpinb6b repressed their expression (Figure 3G,H).

### 3.4 Bmp2 regulation of Serpinb6b expression

**FIGURE 4:**
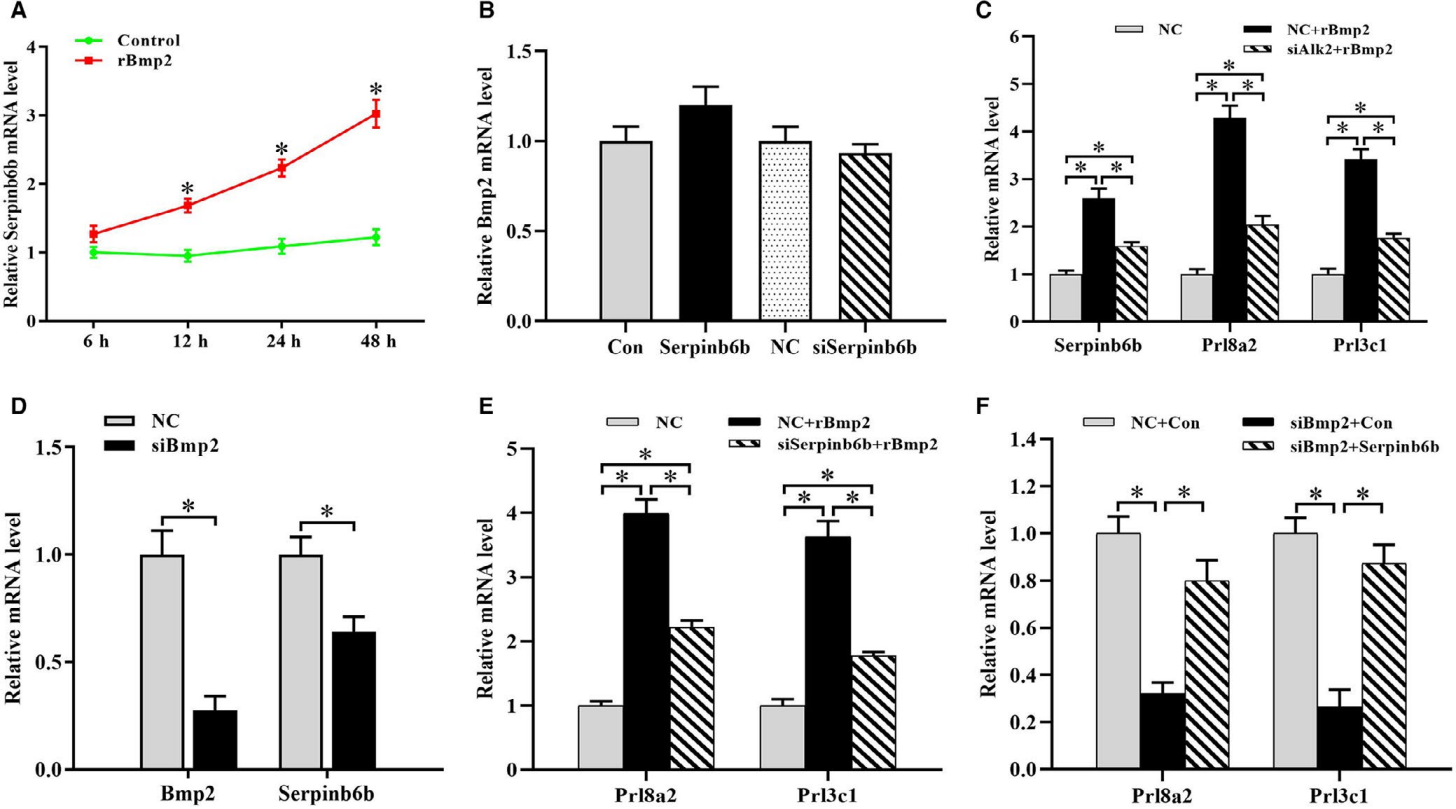
Bmp2 regulation of Serpinb6b expression. A, Serpinb6b expression after treatment with rBmp2 for 6, 12, 24 and 48 hours. B, Effects of Serpinb6b overexpression or silencing on Bmp2 expression. C, Alk2 siRNA blocked the rBmp2 regulation of Serpinb6b, Prl8a2 and Prl3c1. D, Effects of Bmp2 siRNA on Bmp2 and Serpinb6b expression. E, Serpinb6b silencing inhibited the rBmp2 induction of Prl8a2 and Prl3c1. F, Serpinb6b overexpression reversed the suppression of Bmp2 siRNA on Prl8a2 and Prl3c1 expression. siAlk2, Alk2 siRNA; siBmp2, Bmp2 siRNA. *denote significance (*P* < .05)

As Bmp2 is intensively expressed in uterine decidua of pregnant mice and has been demonstrated to be a key regulator of decidualization,^5^, ^6^, ^7^, ^8^, ^22^ we studied the relevance between Bmp2 and Serpinb6b. After exposure to rBmp2, stromal differentiation was induced followed by a time‐dependent elevation for Serpinb6b expression (Figure 4A; Figure S2A). Moreover, neither overexpression nor silencing of Serpinb6b had obvious effects on Bmp2 expression (Figure 4B). When activin‐like kinase 2 (Alk2), a Bmp type I receptor, was silenced by specific siRNA, the up‐regulation of rBmp2 on Serpinb6b expression was significantly weakened, accompanying with reduced mRNA levels of Prl8a2 and Prl3c1 (Figure 4C). By contrast, knock‐down of Bmp2 had the opposite effectiveness (Figure 4D; Figure S2B). To verify whether Serpinb6b was involved in Bmp2 function in stromal differentiation, we transfected the stromal cells with Serpinb6b siRNA, then added rBmp2 and analysed the expression of Prl8a2 and Prl3c1. The results showed that attenuation of Serpinb6b expression greatly reduced the rBmp2 induction of Prla8a2 and Prl3c1 (Figure 4E). Conversely, overexpression of Serpinb6b evidently reversed the suppression of Prl8a2 and Prl3c1 by Bmp2 siRNA (Figure 4F).

### 3.5 Serpinb6b mediated the effects of cAMP on stromal cell differentiation

**FIGURE 5:**
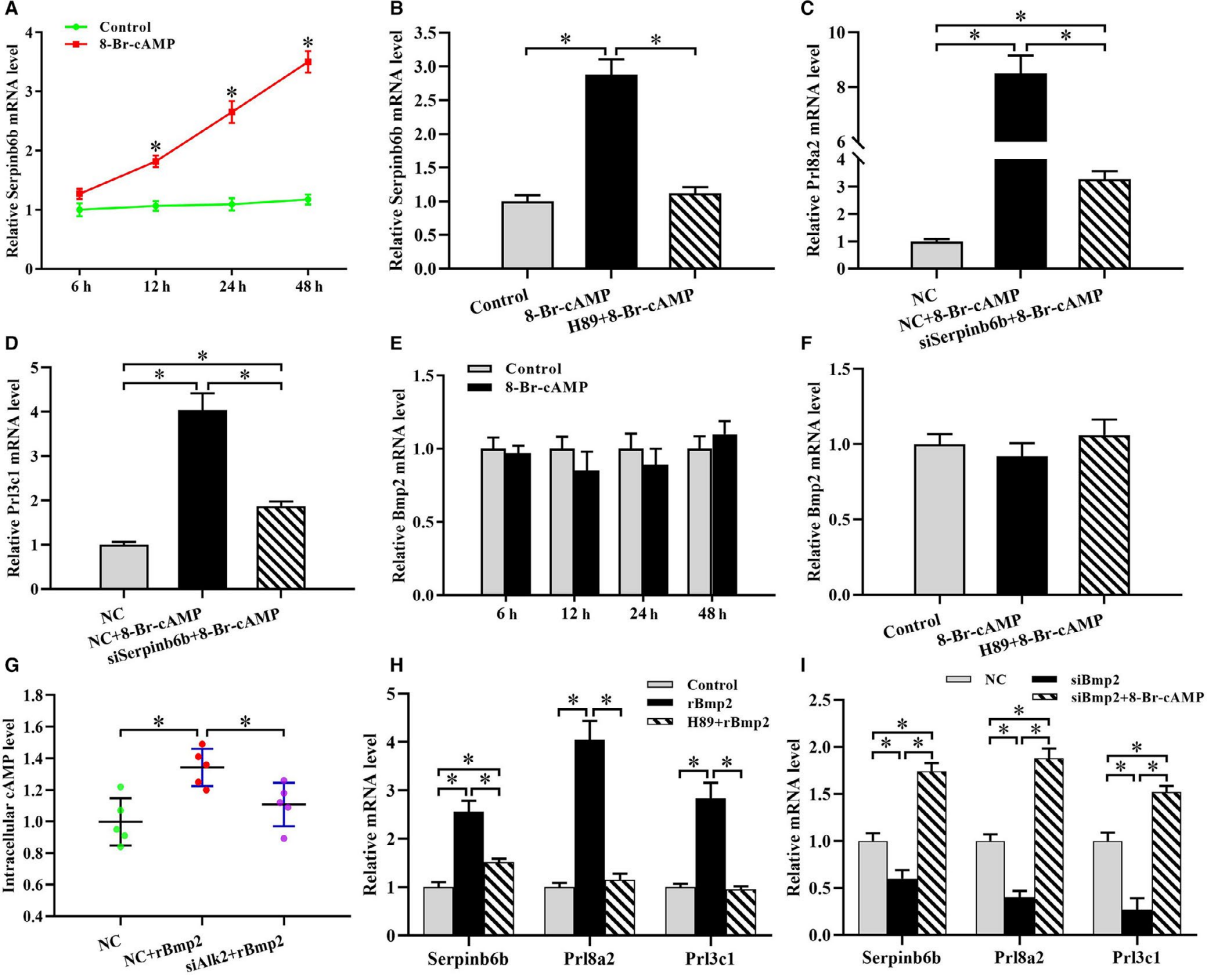
Bmp2 regulates Serpinb6b expression via cAMP‐PKA signalling. A, Serpinb6b expression after treatment with 8‐Br‐cAMP for 6, 12, 24 and 48 h. B, Induction of Serpinb6b expression by 8‐Br‐cAMP was abrogated by H89. C and D, Knock‐down of Serpinb6b inhibited the 8‐Br‐cAMP induction of Prl8a2 and Prl3c1. E and F, Bmp2 expression after exposure to 8‐Br‐cAMP for 6, 12, 24 and 48 h with/without H89. G, Effects of rBmp2 on intracellular cAMP level in the absence or presence of Alk2 siRNA. H, H89 impeded the rBmp2 induction of Serpinb6b, Prl8a2 and Prl3c1. I, 8‐Br‐cAMP prevented the repression of Bmp2 siRNA on Serpinb6b, Prl8a2 and Prl3c1 expression. *denote significance (*P* < .05)

Activation of cAMP‐PKA signalling is vital for induction of decidualization.^23^, ^24^ In uterine stromal cells, Serpinb6b expression was significantly up‐regulated by cAMP analogue 8‐Br‐cAMP treatment in a time‐dependent manner (Figure 5A). However, this up‐regulation was completely blocked by protein kinase A (PKA) inhibitor H89 (Figure 5B). We next determined whether Serpinb6b exerted a role in cAMP‐mediated stromal differentiation. The results showed that silencing of Serpinb6b impeded the enhancement of Prl8a2 and Prl3c1 expression elicited by 8‐Br‐cAMP (Figure 5C,D).

### 3.6 Bmp2 regulates Serpinb6b expression via cAMP‐PKA signalling

In stromal cells, 8‐Br‐cAMP did not adjust Bmp2 expression with or without PKA inhibitor H89 (Figure 5E,F). However, supplementation of exogenous rBmp2 resulted in an obvious accumulation of intracellular cAMP level, but this accumulation was reversed by Alk2 siRNA (Figure 5G). In the meantime, H89 impeded the induction of stromal differentiation by rBmp2, whereas the differentiation defects caused by Bmp2 knock‐down were improved by 8‐Br‐cAMP, implying that Bmp2 is upstream of cAMP‐PKA signalling (Figure 5H,I). As demonstrated above, Serpinb6b was regulated by cAMP via PKA signalling. We next dissected whether Bmp2 regulation of Serpinb6b was mediated by cAMP‐PKA signalling. After pre‐treatment with H89, the induction of rBmp2 on Serpinb6b was remarkably blocked (Figure 5H). In contrast, 8‐Br‐cAMP ameliorated the reduction of Serpinb6b generated by Bmp2 knock‐down (Figure 5I).

### 3.7 Wnt4 mediates the regulation of cAMP on Serpinb6b expression

**FIGURE 6:**
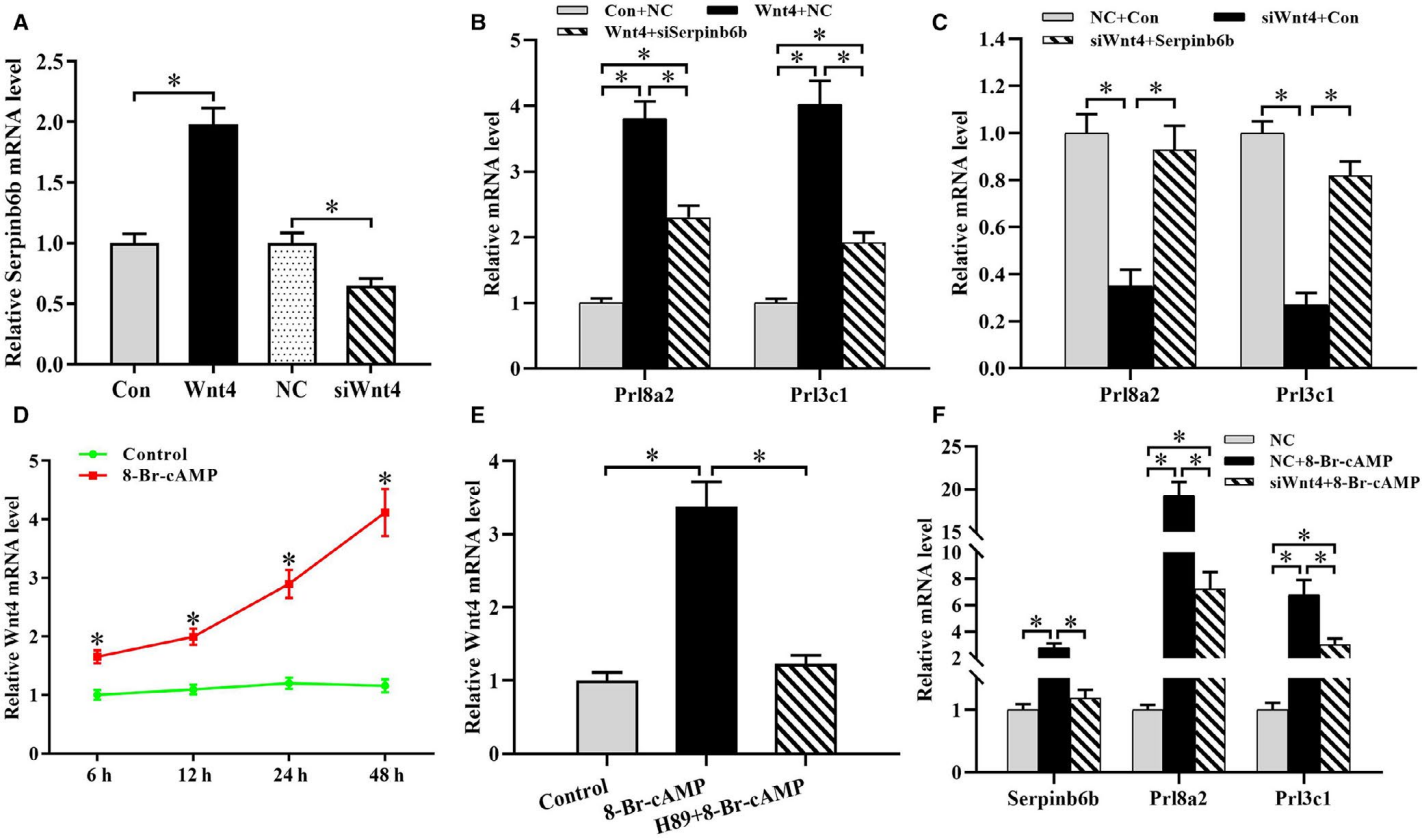
Wnt4 mediates the regulation of cAMP‐PKA signalling on Serpinb6b. A, Effects of Wnt4 overexpression or silencing on Serpinb6b expression. B, Knock‐down of Serpinb6b impeded the induction of Wnt4 overexpression on Prl8a2 and Prl3c1 expression. C, Serpinb6b overexpression prevented the suppression of Wnt4 siRNA on Prl8a2 and Prl3c1 expression. D, Wnt4 expression after exposure to 8‐Br‐cAMP. E, H89 abrogated the stimulation of Wnt4 expression by 8‐Br‐cAMP. F, Wnt4 mediated the cAMP regulation of Serpinb6b, Prl8a2 and Prl3c1. Wnt4, Wnt4 overexpression plasmid; siWnt4, Wnt4 siRNA. *denote significance (*P* < .05)

Wnt4 is important for uterine decidualization.^5^, ^7^, ^25^ Sustained activation of Wnt4 resulted in a dramatic up‐regulation in Serpinb6b expression along with the elevation of Wnt4, Prl8a2 and Prl3c1 mRNA levels, while knock‐down of Wnt4 displayed the overturned influences (Figure 6A; Figure S2C,D). Concurrently, there was no obvious alteration for Wnt4 mRNA level after transfection with Serpinb6b overexpression plasmid or siRNA (Data not shown). Furthermore, blockage of Serpinb6b hampered the up‐regulation of Prl8a2 and Prl3c1 induced by Wnt4 overexpression (Figure 6B). Conversely, overexpression of Serpinb6b could rescue the defects of stromal differentiation by Wnt4 silencing (Figure 6C).

In uterine stromal cells, 8‐Br‐cAMP could significantly raise Wnt4 expression via PKA signalling (Figure 6D,E). Moreover, repression of Wnt4 impeded the induction of stromal cell differentiation accompanying with a lost elevation of Serpinb6b expression yielded by cAMP (Figure 6F).

### 3.8 Bmp2 modulates Serpinb6b expression through cAMP/PKA/Wnt4 pathway

**FIGURE 7:**
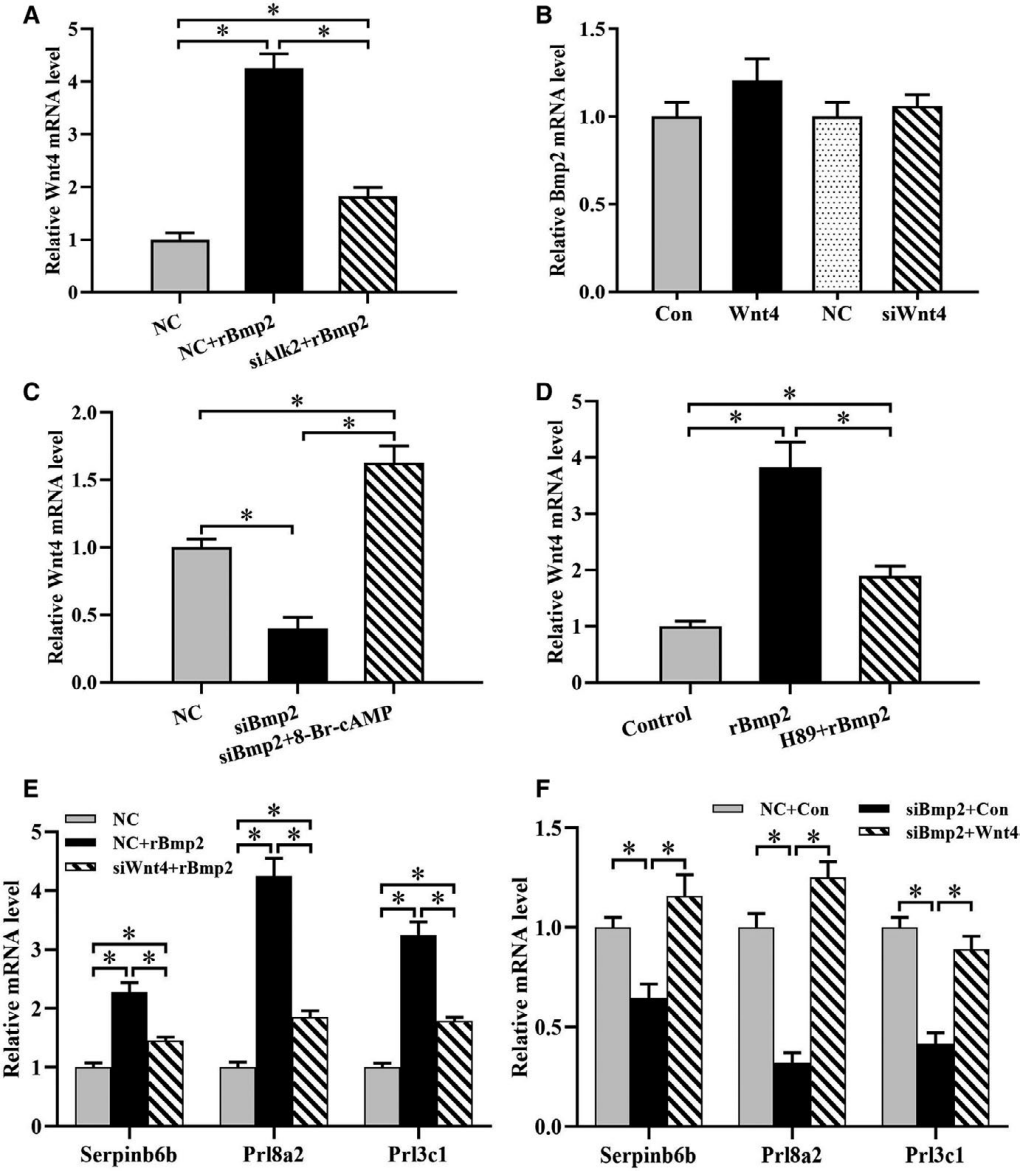
Bmp2 modulates Serpinb6b expression through cAMP/PKA/Wnt4 pathway. A, Alk2 siRNA impeded the induction of Wnt4 expression by rBmp2. B, Effects of Wnt4 overexpression or silencing on Bmp2 expression. C, 8‐Br‐cAMP reversed the inhibition of Bmp2 siRNA on Wnt4 expression. D, H98 blocked the rBmp2 induction of Wnt4. E, Wnt4 knock‐down attenuated the rBmp2 stimulation of Serpinb6b, Prl8a2 and Prl3c1. F, Wnt4 overexpression improved the repression of Bmp2 siRNA on Serpinb6b, Prl8a2 and Prl3c1. *denote significance (*P* < .05)

After supplementation of exogenous rBmp2, Wnt4 induction was noted in stromal cells, but this induction was hindered by Alk2 siRNA (Figure 7A). Both overexpression and inhibition of Wnt4 did not change the expression of Bmp2 in the stromal cells (Figure 7B). As reported previously, Alk2 induced the accumulation of intracellular cAMP level.^19^ Addition of 8‐Br‐cAMP rescued the suppression of Bmp2 siRNA on Wnt4 expression, whereas PKA inhibitor H89 treatment led to a failure in the stimulation of rBmp2 on Wnt4 (Figure 7C,D).

As described above, Bmp2 regulated the expression of Serpinb6b through cAMP‐PKA signalling which was upstream of Wnt4. We next elucidated that Wnt4 might play a role in Bmp2 regulation on Serpinb6b. The results declared that knock‐down of Wnt4 prevented the rBmp2 stimulation on Serpinb6b, while constitutively activated Wnt4 reformed the down‐regulation of Serpinb6b elicited by Bmp2 siRNA (Figure 7E,F). Additionally, Wnt4 might mediate the Bmp2 effects on stromal differentiation (Figure 7E,F).

## 4 DISCUSSION

Serpinb6b is a novel identified member of Serpinb family, but its biology function remains unclear although was found in germ and somatic cells of mouse gonads.^16^ The present study revealed that abundant Serpinb6b was noted in decidual cells, which confirmed the previous microarray data,^17^ suggesting an involvement of Serpinb6b in uterine decidualization.

It has been established that uterine decidualization is characterized by extensive proliferation and differentiation of stromal cells in mice.^2^ Here, Serpinb6b could enhance the proliferation activity of uterine stromal cells. It is well known that cyclins and Cdks are the main regulator of mammalian cell proliferation.^26^ Ccna1 has been demonstrated to be involved in the cell cycle progression from S to G2 phase.^26^ Among the numerous Cdks, Cdk1 is truly essential for mammalian cell cycle because disruption of Cdk1 is embryonic lethal at blastocyst stage, while mice deficiency of Cdk2, Cdk3, Cdk4 or Cdk6 are viable.^27^, ^28^ Previous studies reported that Ccna1 was able to complex with Cdk1 to drive the S to G2 phase transition and also coordinate with Cdk2 to facilitate cell entry into the S phase.^29^, ^30^ In this study, Serpinb6b could induce the expression of Ccna1 and Cdk1, but had no effect on the expression of Cdk2, implying that Serpinb6b is involved in regulating cell cycle progression from S into G2 phase.

Differentiation of stromal cells into polyploidy decidual cells is a key event for continued embryonic development and successful pregnancy.^2^, ^8^ Prl8a2 and Prl3c1 levels, the reliable markers for uterine stromal cell differentiation during decidualization,^19^, ^20^ were significantly increased by overexpression of Serpinb6b, suggesting an importance of Serpinb6b in stromal differentiation. Meanwhile, uterine decidualization involved in extracellular matrix remodelling that was mediated by matrix metalloproteinases whose blockage might reduce the length and size of the deciduas.^31^, ^32^ In stromal cells, Serpinb6b induced the expression of Mmp2 and Mmp9, implying Serpinb6b involvement in uterine extracellular matrix remodelling during decidualization.

It has been established that Bmp2 is an important regulator of stromal cell differentiation in mice and humans.^5^, ^7^, ^8^ Bmp2 was sufficient to endometrial decidualization, because its deficiency led to defective stromal differentiation.^5^, ^7^, ^8^ Treatment with rBmp2 could promote the expression of Serpinb6b. Moreover, overexpression of Serpinb6b significantly rescued the reduction of Prl8a2 and Prl3c1 expression by Bmp2 knock‐down, while down‐regulation of Serpinb6b caused the failure of rBmp2 in promoting stromal differentiation. Taken together, these findings strongly imply that Serepinb6b may be a downstream target of Bmp2 during decidualization. Furthermore, Bmp2 regulation of Serepinb6b was blocked by BMP type I receptor Alk2 which induced the accumulation of intracellular cAMP level.^19^

Activation of cAMP‐PKA signalling is a prerequisite for uterine decidualization.^23^, ^24^ In stromal cells, cAMP analogue 8‐Br‐cAMP significantly elevated the expression of Serpinb6b via PKA signalling. Suppression of Serpinb6b retarded the induction of 8‐Br‐cAMP on stromal differentiation. It has previously reported that cAMP enhanced the Bmp2‐induced osteoblast differentiation, and PKA activation is pivotal in Bmp2‐mediated chondrogesis.^33^, ^34^ These observations suggest that cAMP‐PKA signalling is downstream of Bmp2, which is further strengthened in decidualization. Further analysis found that PKA inhibitor H89 blocked the promotion of Bmp2 on Serpinb6b, while 8‐Br‐cAMP rescued the fault of Serpinb6b elicited by Bmp2 knock‐down, indicating that cAMP‐PKA signalling exerts a role in the connection between Bmp2 and Serpinb6b.

It is generally accepted that Wnt4 is a crucial regulator of stromal cell decidualization.^7^, ^8^, ^25^ Conditional ablation of Wnt4 in mouse uterus led to the failed decidual response.^25^ The present study has identified Serpinb6b as a target of Wnt4 regulation in stromal differentiation. Previous evidence demonstrated that Wnt4 functioned as downstream of Bmp2 in uterine decidualization.^7^, ^8^ In this study, we found that cAMP‐PKA signalling was involved in the regulation of Bmp2 on Wnt4. Furthermore, Bmp2 might modulate the expression of Serpinb6b via cAMP/PKA/Wnt4 pathway.

**FIGURE 8:**
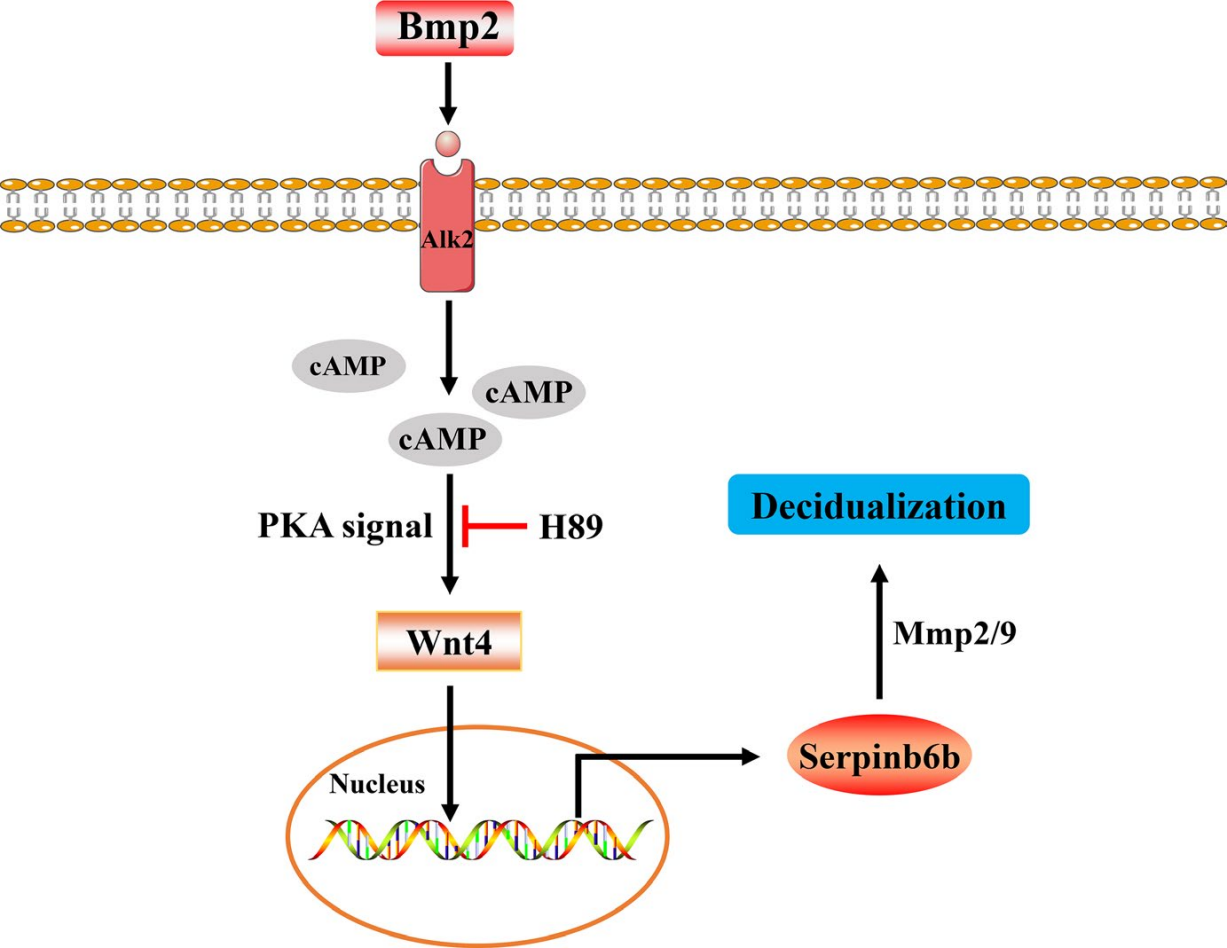
Schematic illustration of Serpinb6b regulation in uterine decidualization. Serpinb6b might mediate uterine decidualization via Mmp2/9 and act as a downstream target of cAMP/PKA/Wnt4 pathway in response to Bmp2

In conclusion, the present study revealed a novel insight into the role of Serpinb6b in uterine decidualization via Mmp2/9 and identified it as a downstream target of Bmp2/cAMP/PKA/Wnt4 pathway (Figure 8).

## CONFLICT OF INTEREST

The authors confirm that there are no conflicts of interest.

## Supporting information

Figure S1

Figure S2

## Data Availability

The data that support the findings of this study are available on request from the corresponding author.

## References

[1] Ramathal CY , Bagchi IC , Taylor RN , et al. Endometrial decidualization: of mice and men. Semin Reprod Med. 2010;28:17‐26. 20104425 10.1055/s-0029-1242989 PMC3095443

[2] Zhang S , Lin H , Kong S , et al. Physiological and molecular determinants of embryo implantation. Mol Aspects Med. 2013;34:939‐980. 23290997 10.1016/j.mam.2012.12.011 PMC4278353

[3] Salker M , Teklenburg G , Molokhia M , et al. Natural selection of human embryos: impaired decidualization of endometrium disables embryo‐maternal interactions and causes recurrent pregnancy loss. PLoS One. 2010;5:e10287. 20422017 10.1371/journal.pone.0010287 PMC2858209

[4] Kommagani R , Szwarc MM , Kovanci E , et al. Acceleration of the glycolytic flux by steroid receptor coactivator‐2 is essential for endometrial decidualization. PLoS Genet. 2013;9:e1003900. 24204309 10.1371/journal.pgen.1003900 PMC3812085

[5] Lee KY , Jeong JW , Wang J , et al. Bmp2 is critical for the murine uterine decidual response. Mol Cell Biol. 2007;27:5468‐5478. 17515606 10.1128/MCB.00342-07 PMC1952078

[6] Nallasamy S , Kaya Okur HS , Bhurke A , et al. Msx homeobox genes act downstream of BMP2 to regulate endometrial decidualization in mice and in humans. Endocrinology. 2019;160:1631‐1644. 31125045 10.1210/en.2019-00131 PMC6591014

[7] Li Q , Kannan A , Wang W , et al. Bone morphogenetic protein 2 functions via a conserved signaling pathway involving Wnt4 to regulate uterine decidualization in the mouse and the human. J Biol Chem. 2007;282:31725‐31732. 17711857 10.1074/jbc.M704723200

[8] Li Q , Kannan A , Das A , et al. WNT4 acts downstream of BMP2 and functions via β‐catenin signaling pathway to regulate human endometrial stromal cell differentiation. Endocrinology. 2013;154:446‐457. 23142810 10.1210/en.2012-1585 PMC3529366

[9] Heit C , Jackson BC , McAndrews M , et al. Update of the human and mouse SERPIN gene superfamily. Hum Genomics. 2013;7:22. 24172014 10.1186/1479-7364-7-22 PMC3880077

[10] Vidalino L , Doria A , Quarta S , et al. SERPINB3, apoptosis and autoimmunity. Autoimmun Rev. 2009;9:108‐112. 19332150 10.1016/j.autrev.2009.03.011

[11] Loison F , Zhu H , Karatepe K , et al. Proteinase 3‐dependent caspase‐3 cleavage modulates neutrophil death and inflammation. J Clin Invest. 2014;124:4445‐4458. 25180606 10.1172/JCI76246 PMC4191030

[12] Rangel R , Lee SC , Hon‐Kim Ban K , et al. Transposon mutagenesis identifies genes that cooperate with mutant Pten in breast cancer progression. Proc Natl Acad Sci U S A. 2016;113:E7749‐E7758. 27849608 10.1073/pnas.1613859113 PMC5137755

[13] Riaz N , Havel JJ , Kendall SM , et al. Recurrent SERPINB3 and SERPINB4 mutations in patients who respond to anti‐CTLA4 immunotherapy. Nat Genet. 2016;48:1327‐1329. 27668655 10.1038/ng.3677 PMC5553281

[14] Burgener SS , Leborgne NGF , Snipas SJ , et al. Cathepsin G inhibition by serpinb1 and serpinb6 prevents programmed necrosis in neutrophils and monocytes and reduces GSDMD‐driven inflammation. Cell Rep. 2019;27:3646‐3656. 31216481 10.1016/j.celrep.2019.05.065 PMC7350907

[15] Choi YJ , Kim S , Choi Y , et al. SERPINB1‐mediated checkpoint of inflammatory caspase activation. Nat Immunol. 2019;20:276‐287. 30692621 10.1038/s41590-018-0303-z PMC6450391

[16] Charron Y , Madani R , Nef S , et al. Expression of serpinb6 serpins in germ and somatic cells of mouse gonads. Mol Reprod Dev. 2006;73:9‐19. 16175637 10.1002/mrd.20385

[17] Ma X , Gao F , Rusie A , et al. Decidual cell polyploidization necessitates mitochondrial activity. PLoS One. 2011;6:e26774. 22046353 10.1371/journal.pone.0026774 PMC3201964

[18] Yu HF , Tao R , Yang ZQ , et al. Ptn functions downstream of C/EBPβ to mediate the effects of cAMP on uterine stromal cell differentiation through targeting Hand2 in response to progesterone. J Cell Physiol. 2018;233:1612‐1626. 28657144 10.1002/jcp.26067

[19] Yu HF , Yue ZP , Wang K , et al. Gja1 acts downstream of Acvr1 to regulate uterine decidualization via Hand2 in mice. J Endocrinol. 2017;233:145‐157. 28219934 10.1530/JOE-16-0583

[20] Clementi C , Tripurani SK , Large MJ , et al. Activin‐like kinase 2 functions in peri‐implantation uterine signaling in mice and humans. PLoS Genet. 2013;9:e1003863. 24244176 10.1371/journal.pgen.1003863 PMC3828128

[21] Yue L , Yu HF , Yang ZQ , et al. Egr2 mediates the differentiation of mouse uterine stromal cells responsiveness to HB‐EGF during decidualization. J Exp Zool B Mol Dev Evol. 2018;330:215‐224. 29781132 10.1002/jez.b.22807

[22] Paria BC , Ma W , Tan J , et al. Cellular and molecular responses of the uterus to embryo implantation can be elicited by locally applied growth factors. Proc Natl Acad Sci U S A. 2001;98:1047‐1052. 11158592 10.1073/pnas.98.3.1047 PMC14706

[23] Gellersen B , Brosens J . Cyclic AMP and progesterone receptor cross‐talk in human endometrium: a decidualizing affair. J Endocrinol. 2003;178:357‐372. 12967329 10.1677/joe.0.1780357

[24] Liang XH , Deng WB , Liu YF , et al. Non‐coding RNA LINC00473 mediates decidualization of human endometrial stromal cells in response to cAMP signaling. Sci Rep. 2016;6:22744. 26947914 10.1038/srep22744 PMC4780002

[25] Franco HL , Dai D , Lee KY , et al. WNT4 is a key regulator of normal postnatal uterine development and progesterone signaling during embryo implantation and decidualization in the mouse. FASEB J. 2011;25:1176‐1187. 21163860 10.1096/fj.10-175349 PMC3058697

[26] Das SK . Cell cycle regulatory control for uterine stromal cell decidualization in implantation. Reproduction. 2009;137:889‐899. 19307426 10.1530/REP-08-0539

[27] Diril MK , Ratnacaram CK , Padmakumar VC , et al. Cyclin‐dependent kinase 1 (Cdk1) is essential for cell division and suppression of DNA re‐replication but not for liver regeneration. Proc Natl Acad Sci U S A. 2012;109:3826‐3831. 22355113 10.1073/pnas.1115201109 PMC3309725

[28] Santamaría D , Barrière C , Cerqueira A , et al. Cdk1 is sufficient to drive the mammalian cell cycle. Nature. 2007;448:811‐815. 17700700 10.1038/nature06046

[29] Malumbres M , Barbacid M . Cell cycle, CDKs and cancer: a changing paradigm. Nat Rev Cancer. 2009;9:153‐166. 19238148 10.1038/nrc2602

[30] Otto T , Sicinski P . Cell cycle proteins as promising targets in cancer therapy. Nat Rev Cancer. 2017;17:93‐115. 28127048 10.1038/nrc.2016.138 PMC5345933

[31] Dey SK , Lim H , Das SK , et al. Molecular cues to implantation. Endocr Rev. 2004;25:341‐373. 15180948 10.1210/er.2003-0020

[32] Chen L , Belton RJ Jr , Nowak RA . Basigin‐mediated gene expression changes in mouse uterine stromal cells during implantation. Endocrinology. 2009;150:966‐976. 18832103 10.1210/en.2008-0571 PMC2646530

[33] Lee YS , Chuong CM . Activation of protein kinase A is a pivotal step involved in both BMP‐2‐ and cyclic AMP‐induced chondrogenesis. J Cell Physiol. 1997;170:153‐165. 9009144 10.1002/(SICI)1097-4652(199702)170:2<153::AID-JCP7>3.0.CO;2-N

[34] Ghayor C , Ehrbar M , San Miguel B , et al. cAMP enhances BMP2‐signaling through PKA and MKP1‐dependent mechanisms. Biochem Biophys Res Commun. 2009;381:247‐252. 19217886 10.1016/j.bbrc.2009.02.032

