# Supplementary figures and images for: Bmp2 regulates Serpinb6b expression via cAMP/PKA/Wnt4 pathway during uterine decidualization
Source: J Cell Mol Med. 2020 May 11;24(12):7023–33. doi: 10.1111/jcmm.15372 (PMC7299730; doi:10.1111/jcmm.15372)

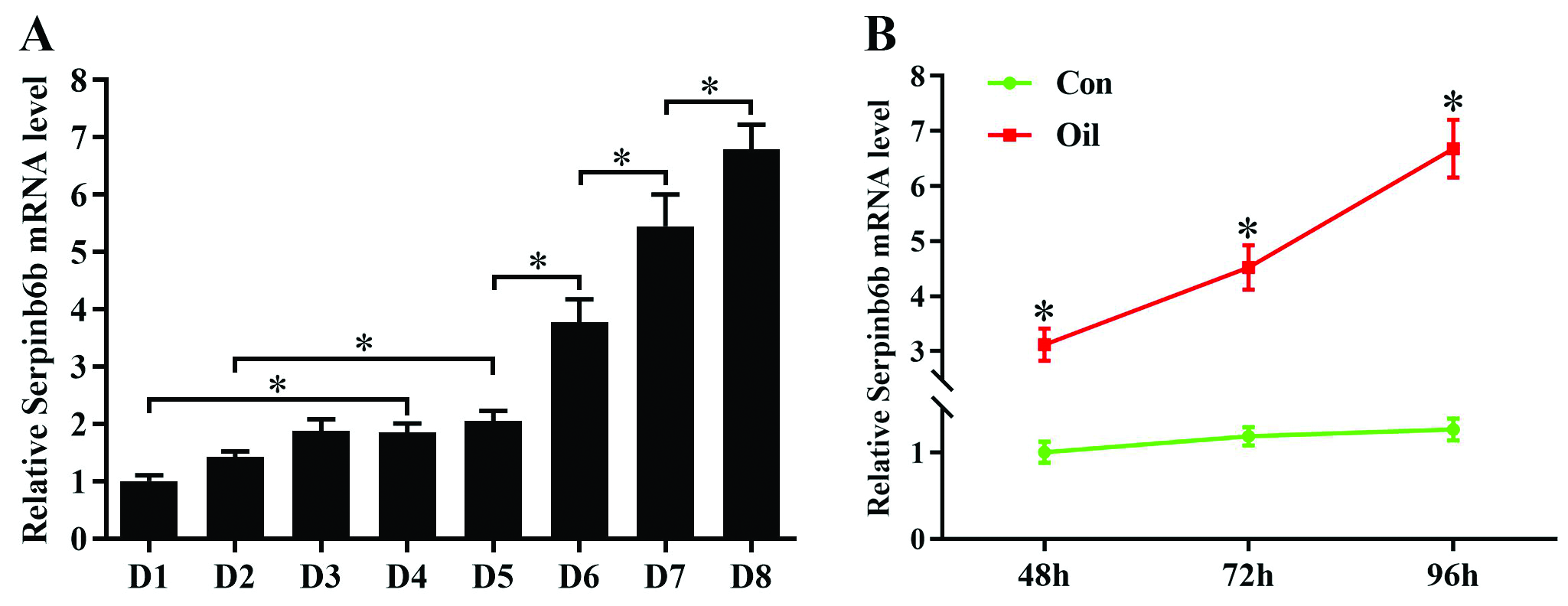

Supplement: Supplementary file 1 — Figure S1 [file JCMM-24-7023-s001.TIF]

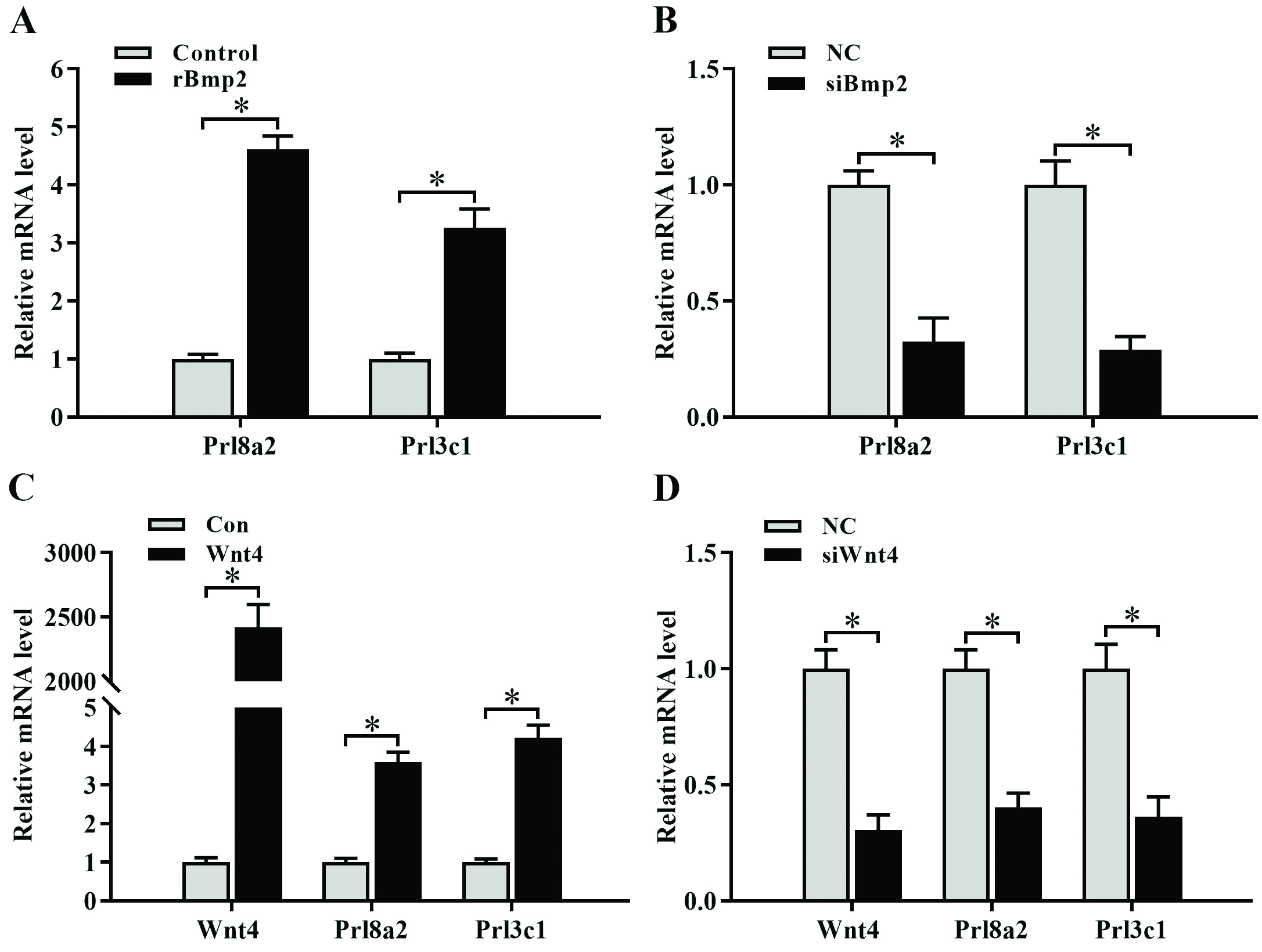

Supplement: Supplementary file 2 — Figure S2 [file JCMM-24-7023-s002.TIF]
